# A Comparison of DSM-IV-TR and DSM-5 Diagnostic Criteria for Gambling Disorder in a Large Clinical Sample

**DOI:** 10.3389/fpsyg.2019.00931

**Published:** 2019-04-26

**Authors:** Susana Jiménez-Murcia, Roser Granero, Fernando Fernández-Aranda, Anne Sauvaget, Andreas Fransson, Anders Hakansson, Gemma Mestre-Bach, Trevor Steward, Randy Stinchfield, Laura Moragas, Neus Aymamí, Mónica Gómez-Peña, Amparo del Pino-Gutiérrez, Zaida Agüera, Marta Baño, Maria-Teresa Talón-Navarro, Àngel Cuquerella, Ester Codina, José M. Menchón

**Affiliations:** ^1^Department of Psychiatry, Bellvitge University Hospital-IDIBELL, Barcelona, Spain; ^2^Ciber Fisiopatología de la Obesidad y Nutrición (CIBERobn), Instituto de Salud Carlos III, Madrid, Spain; ^3^Department of Clinical Sciences, School of Medicine and Health Sciences, University of Barcelona, Barcelona, Spain; ^4^Departament de Psicobiologia i Metodologia de les Ciències de la Salut, Universitat Autònoma de Barcelona, Barcelona, Spain; ^5^Addictology and Liaison Psychiatry Department, Nantes University Hospital, Nantes, France; ^6^Faculty of Medicine, Department of Clinical Sciences Psychiatry, Lund, Sweden; ^7^Department of Psychiatry, University of Minnesota Medical School, Saint Paul, MN, United States; ^8^Department of Public Health, Mental Health and Mother-Infant Nursing, University School of Nursing, University of Barcelona, Barcelona, Spain; ^9^Institute of Legal Medicine and Forensic Sciences of Catalonia, Barcelona, Spain; ^10^CIBER Salud Mental (CIBERSAM), Instituto de Salud Carlos III, Madrid, Spain

**Keywords:** DSM-IV-TR, DSM-5, gambling disorder, criminal behaviors, severity, personality, psychopathology

## Abstract

**Background and Aims:** Gambling-related crimes are known to be associated with gambling disorder (GD). Due to a lack of consensus in the scientific community regarding the relevance of this diagnostic criterion, it was removed from the DSM-5. The primary aim of this study was to investigate through structural equation modeling (SEM) whether higher GD severity in treatment-seeking GD patients with a criminal record is mediated through the illegal acts criterion itself, or whether it can be better explained by other related clinical factors.

**Methods:** An initial sample of 2,081 patients seeking treatment for gambling problems was included in the sample. SEM was used to evaluate the mediational role of the illegal acts criterion between the sex, age and personality traits, gambling severity, and comorbid depression levels. Comparisons between patients with coinciding and divergent DSM criterion for GD diagnosis were carried out.

**Results:** Illegal acts mediated the relationship between personality traits and GD severity: younger age, high levels of novelty seeking, and low levels of self-transcendence increased the risk of endorsing the illegal acts criterion. No differences between coincident-divergent groups in terms of DSM-IV and DSM-5 diagnosis were found with regards to sex (*p* = 0.878), education level (*p* = 0.387), or civil status (*p* = 0.792).

**Discussion and Conclusion:** The results obtained in the present study offer new insights into the utility of using a history of illegal acts, their different personality characteristics, and psychopathology to categorize GD patients. Our findings suggest that patients who engage in criminal behavior may require a more comprehensive intervention.

## Introduction

Gambling-related crimes are known to be associated with gambling disorder (GD), allowing individuals with this disorder to obtain funding to continue with the gambling behavior or to solve financial problems stemming from it (Petry et al., 2013; Laursen et al., 2016; May-Chahal et al., 2017; Mestre-Bach et al., 2018a). Some risk factors, such as sociodemographic features (Abbott and McKenna, 2005), personality disorders (e.g., antisocial personality disorder) and traits (Turner et al., 2009; Pastwa-Wojciechowska, 2011) and the co-occurrence of substance use (Gorsane et al., 2017) have been highlighted when taking GD, and criminal behavior into account. All these factors would be associated with a pattern of behavior characterized by a greater risk-taking (Bornovalova et al., 2005; Adolphe et al., 2018).

The DSM-IV-TR, therefore, included a criterion focused on these illegal acts carried out for gambling-related reasons, specifically assessing whether the subject “has committed illegal acts such as forgery, fraud, theft, or embezzlement to finance gambling” (American Psychiatric Association, 2000). Nevertheless, the more recent version of the DSM-5 and, more specifically, the Pathological Gambling Committee opted to both lower the cut-off score from five to four and to remove the illegal acts criterion (American Psychiatric Association, 2013). These two changes were completely separate and were not related to each other. Research related to the removal of the illegal acts criterion have demonstrated that this has had little impact on the number of patients being diagnosed with the disorder, that the illegal acts criterion was the criterion least commonly endorsed by patients, and that patients who met this criterion in the DSM-IV-TR generally also met a sufficient number of other criteria to meet diagnosis without the presence of illegal acts (Zimmerman et al., 2006; Strong and Kahler, 2007; McBride et al., 2010; Weinstock et al., 2013; Granero et al., 2014). Moreover, most researchers consider it should not have been categorized as an independent diagnostic criterion, but as an indicator of GD severity (Jiménez-Murcia et al., 2009; Petry et al., 2013; Granero et al., 2014). Likewise, the cut-off for the diagnosis of this disorder was changed from five to four criteria, based on a series of studies published in the early 2000s showing that this change improved classification accuracy and reduced the false negative rate from 0.08 to 0.05 (Stinchfield, 2003; Jiménez-Murcia et al., 2009).

The implications of omitting the illegal acts criterion in the general population have been evaluated, most significantly in the National Epidemiological Survey of Alcohol and Related Conditions, using a large American study sample (Petry et al., 2013). However, studies have shown that treatment-seeking GD patients tend to report the illegal acts criterion at much higher proportions than compared to population-based samples of ordinary gamblers (Toce-Gerstein et al., 2003; Petry et al., 2013; Granero et al., 2014). Still, research targeting possible consequences of removing this criterion in a clinical setting of treatment-seeking patients has up to now been scarce or based on patient populations seeking treatment for any addictive disorder (Denis et al., 2012; Petry et al., 2013).

Data from clinical samples have demonstrated that patients with GD who endorse the illegal acts criterion show higher GD severity at baseline and throughout treatment, indicating that this clinical group may need further interventions (Potenza et al., 2000; Strong and Kahler, 2007; Mestre-Bach et al., 2018a,b). Correspondingly, Turner et al. (2016) demonstrated the relevance of assessing criminal behavior in problematic gamblers in a criminal justice population.

In order to delve into the repercussions of this change at a clinical level, the primary aim of the present study was to investigate through path analysis implemented in structural equation modeling (SEM) whether higher GD severity seen in treatment-seeking GD patients with a criminal history is mediated through the illegal acts criterion itself, or whether it can be better explained by other related clinical factors.

## Materials and Methods

### Participants and Procedure

The sample included 2,081 patients seeking treatment for gambling problems from the Department of Psychiatry at Bellvitge University Hospital in Barcelona, Spain. Diagnosis was carried out according to DSM-IV-TR criteria (American Psychiatric Association, 2000), through the Stinchfield diagnostic questionnaire, based on the DSM-IV criteria (Stinchfield, 2003) adapted and validation to Spanish (Jiménez-Murcia et al., 2009). Diagnoses were made by psychologists and psychiatrists with more than 20 years of clinical experience in the assessment and treatment of GD. Other socio-demographic, clinical, and socio/family variables related to gambling were measured using a semi-structured face-to-face clinical interview. This interview explored motivations for gambling, age of onset of recreational gambling, age of onset of symptoms of problematic, preferred form of gambling (or several if applicable), gambling frequency, maximum bet, average, reinvestment of wins, magical and distorted thinking, gambling-related debts, periods of abstinence, gambling episode behavior, etc.

A total of 152 patients did not meet either DSM-IV criteria or the DSM-5 criteria, and were therefore not considered for further statistical analyses.

Patients included in the study were initially assessed by trained and licensed psychologists and psychiatrists with experience in this behavioral addiction. Information regarding gambling behavior was gathered through a semi-structured face-to-face interview. The assessment was conducted prospectively at baseline in a single session. Patients filled out the SOGS, TCI-R, and SCL-90-R before initiating outpatient treatment.

The present study was carried out in accordance with the latest version of the Declaration of Helsinki. The Bellvitge University Hospital Ethics Clinical Research Committee approved the study. Signed, informed consent was obtained from all patients.

### Measures

#### South Oaks Gambling Screen (SOGS) (Lesieur and Blume, 1987)

The SOGS is a 20-item questionnaire used to screen for GD, predominantly in clinical settings. The total score of the questionnaire is calculated by adding endorsed items, with a total score of 20, where a cut-off score of 5, or more is indicative of probable GD. The Spanish validation of SOGS showed adequate psychometric properties (test-retest reliability, 0.98; internal consistency, 0.94; and convergent validity, 0.92) (Echeburúa et al., 1994). In the present study, only total scale score was used for analyses. Table 1 includes Cronbach’s alpha in the sample of the study.

**Table 1:** SEM results for the pathways valuing the mediational role of illegal acts: standardized coefficients (structural).

		Coeff.	SE	z	*p*	95% CI (coeff.)	
Illegal acts	Age (yrs-old)	-0.155	0.0235	-6.61	<0.001	-0.201	-0.109
	Sex (male)	0.005	0.0230	0.2	0.841	-0.041	0.050
	TCI-R: novelty seeking	0.179	0.0243	7.35	<0.001	0.131	0.226
	TCI-R: self-directedness	-0.114	0.0238	-4.81	<0.001	-0.161	-0.068
	*constant*	0.385	0.3078	1.25	0.21	-0.218	0.989
SOGS-total	Illegal acts	0.194	0.0212	9.14	<0.001	0.152	0.235
	Age (yrs-old)	-0.064	0.0218	-2.02	0.043	-0.087	-0.001
	Sex (male)	0.008	0.0209	0.4	0.693	-0.033	0.049
	TCI-R: novelty seeking	0.258	0.0221	11.66	<0.001	0.215	0.302
	TCI-R: self-directedness	-0.243	0.0213	-11.41	<0.001	-0.285	-0.202
	*constant*	2.710	0.2849	9.51	<0.001	2.152	3.268
Depression	SOGS-total	0.202	0.0205	9.87	<0.001	0.162	0.242
	Age (yrs-old)	0.073	0.0196	3.71	<0.001	0.034	0.111
	Sex (male)	-0.165	0.0194	-8.5	<0.001	-0.203	-0.127
	TCI-R: self-directedness	-0.442	0.0190	-23.3	<0.001	-0.479	-0.405
	*Constant*	3.862	0.1828	21.13	<0.001	3.504	4.220
Covariances	Age, Novelty-seeking	-0.258	0.0221	-11.67	<0.001	-0.301	-0.214
	Age, Sex	-0.176	0.0229	-7.68	<0.001	-0.221	-0.131
	Age, Self-directedness	0.007	0.0237	0.3	0.765	-0.039	0.053
	Novelty-seek., Sex	-0.019	0.0236	-0.82	0.413	-0.066	0.027
	Novelty-seek., Self-direct	-0.317	0.0213	-14.89	<0.001	-0.359	-0.275
	Sex, Self-directedness	0.105	0.0234	4.51	<0.001	0.060	0.151

**Equation level goodness-of-fit**		**Variance fitted**	**Variance predicted**	**Variance residual**	**R^2^**	**mc**	**mc^2^**

Observed	Illegal acts	0.183	0.018	0.166	0.097	0.311	0.097
	SOGS-total score	10.164	2.603	7.562	0.256	0.506	0.256
	Depression	0.817	0.284	0.533	0.348	0.590	0.348

*mc: correlation between dependent variable and its prediction. mc^2^: Bentler-Raykov squared multiple correlation coefficient.*

#### Temperament and Character Inventory-Revised (TCI-R) (Cloninger, 1999)

The TCI-R is a 240-item questionnaire with a five-point Likert scale format used to measure four temperament (harm avoidance, novelty seeking, reward dependence, and persistence) and three character dimensions (self-directedness, cooperativeness, and self-transcendence) of personality. The Spanish adaptation of the TCI-R has showed acceptable reliability of the seven dimensions, with alphas between 0.74 and 0.87 (Gutiérrez-Zotes et al., 2004). For the SEM we only used total scores dimensions that showed a significant association with other indicators analyzed in this study. Table 1 includes Cronbach’s alpha in the study sample.

#### Symptom Checklist-90-R (SCL-90-R) (Derogatis, 1990)

This is a widely used, self-report 90-item inventory measuring psychological distress and psychopathology. The inventory measures nine symptom dimensions: somatization, obsessive-compulsive, interpersonal sensitivity, depression, anxiety, hostility, phobic anxiety, paranoid ideation, and psychoticism. The global score (Global Severity Index [GSI]) is a widely used index of psychopathological distress. The SCL-90-R has been validated in a Spanish population obtaining adequate internal consistency of the items ranging between 0.81 and 0.90, and an acceptable mean internal consistency of 0.75 (Derogatis, 2002). Primary symptom dimensions score were evaluated, but only those that showed a significant association with other indicators analyzed in this study were finally selected for the SEM. Table 1 includes Cronbach’s alpha in the sample of the study.

#### Additional Data

Demographic, clinical, and social/family variables related to gambling were measured using a semi-structured face-to-face clinical interview described elsewhere (Jiménez-Murcia et al., 2006).

### Statistical Analysis

All statistical analyses were carried out with STATA 15 for Windows. Comparison between patients with coinciding and divergent DSM criterion for GD diagnosis was done through chi-square tests for categorical features and *t*-tests for quantitative outcomes. Effect-size of the comparisons was measured through Cohen’s-*d* coefficient, considering |*d*| < 0.50 to be poor effect-size, |*d*| > 0.50 to be moderate, and |*d*| > 0.80 to be high.

The mediational role of the variables of interest was examined through Structural Equation Models. Overall goodness-of-fit statistics were assessed through *χ*^2^ test, the Root Mean Squared Error of Approximation (RMSEA), baseline comparison indexes (Comparative Fit Index [CFI], Tucker-Lewis Index [TLI]), and residuals size (Standardized Mean Squared Residual [SMSR]). Fit was considered good if (Kline, 2011): a non-significant result (*p* > 0.05) was obtained on the *χ*^2^ test, the RMSEA was lower than 0.08, the CFI-TLI coefficients were higher than 0.90 and SRMR was limited to 0.08. The equation level goodness-of-fit and the effect sizes were also estimated through R^2^ coefficients for each equation and for the global model including the set of variables and their relationship pattern (these coefficients valued the fraction of the variance explained by indicator/s), multiple correlation (mc) and Bentler-Raykov multiple correlation (mc^2^ Bentler and Raykov, 2000). These last two coefficients report the relatedness of each dependent variable with the model’s linear prediction (in non-recursive models mc^2^ is computed to avoid the problem of obtaining inconsistent negative mcs).

## Results

### Sample Characteristics

Participants’ mean age was 42.23 years old (*SD* = 13.3), with a mean age of GD onset of 36.26 years (*SD* = 13.2), and a mean GD duration of 5.64 years (*SD* = 6.13). Most participants were male (90.4 %) and had a primary school level of education (57.6%).

Regarding the problematic gambling activities in the sample, 85.9% of the participants reported using slot machines, 12.3% bingo, 9.9% lotteries, 6.1% casinos, 3.9% cards, 3.8% bets on the Internet, and 2.5% bets on sports or horses. The mean number of problematic gambling activities in the sample was 1.3 (*SD* = 0.6; 81.1% of the patients reported a single problematic activity, 13.9% two problematic activity, and the remaining 5.0% at least three problematic activity). Moreover, 76.74% of the sample did not endorse the criminal acts criterion, while 23.26% did. In this vein, more than one third of women and more than half of men reported that they had been involved in illegal acts in order to finance their gambling behavior.

### Mediational Role of Illegal Acts in the Gambling Profile

**FIGURE 1:**
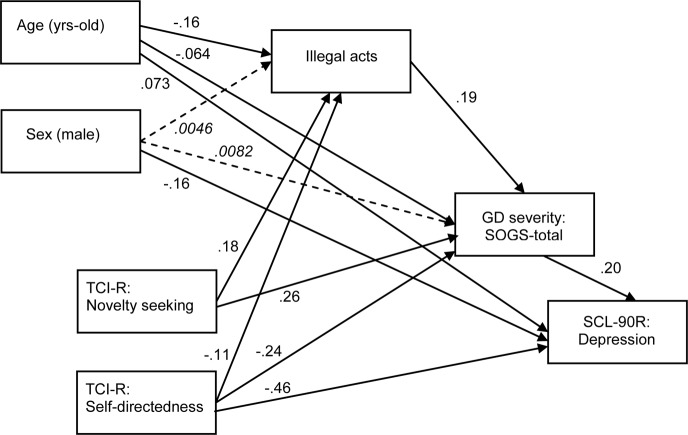
SEM to value the mediational role of illegal acts on gambling severity. Dash-line, italic font: non-significant coefficient.

Table 1 and Figure 1 show the results for the SEM valuing the mediational role of the illegal acts criterion between the sex, age and TCI-R scores, gambling severity (SOGS-total score), and the level of comorbid depression (SCL-90-R, depression score). Goodness-of-fit was obtained: *χ*^2^ = 3.93 (*p* = 0.140), RMSEA = 0.023 (95%CI: 0 to 0.057), CFI = 0.999, TLI = 0.990, and SRMR = 0.006. The global predictive capacity was very good (coefficient of determination CD = 0.447, a measure of the total R^2^ for the model). Illegal acts mediated the relationship between personality traits (age, TCI-R novelty seeking and self-transcendence levels) and GD severity: young age, high levels of novelty seeking, and low levels of self-transcendence increased the risk of endorsing the illegal acts criterion. Furthermore, the presence of the illegal acts criterion predicted higher SOGS-total scores. Age and TCI-R scores also showed a direct effect on GD severity: young age was associated with higher novelty seeking, lower self-transcendence scores, and SOGS-total scores. High levels of depression symptoms (measured by SCL-90-R) were associated with high GD severity, as well as with old age, being female, and low self-transcendence scores.

### Comparison Between Patients With Coincident and Divergent DSM Criteria

In the initial sample of 2,081 patients, two binary conditions were defined according to the DSM-IV and DSM-5 criteria for GD: (a) DSM-IV criteria present versus absent (at least five of the 10 gambling behaviors described in the taxonomy was considered present, including in the count the A8 criterion assessing the presence of illegal acts); and (b) DSM-5 criteria present versus absent (individuals exhibiting at least four of the 9 gambling behaviors described in the taxonomy was considered present, excluding illegal acts). No patient was included in the joint condition DSM-IV = present and DSM-5 = absent.

All remaining GD patients (*n* = 1,929) met the DSM-5_criteria. In this cohort, *n* = 1,822 (94.5%) also met the DSM-IV criteria, resulting in 107 patients (5.5%) not meeting this cut-off (these patients reported exactly four of the maladaptive gambling behaviors described in the DSM). These two groups were labeled as “coinciding DSM_criteria” versus “divergent DSM_criteria” and were compared in terms of sociodemographic features and clinical measures.

No patients in the divergent DSM_criteria group reported illegal acts, while 26% of patients in the coinciding DSM_criteria group endorsed the illegal acts criterion. No difference between coincident-divergent groups was found with regards to sex (*p* = 0.878), education level (*p* = 0.387), or civil status (*p* = 0.792).

The first part of Table 2 contains the results of the comparison between coincident and divergent DSM criteria groups, the presence of other comorbid problems and the substances abuse/use. In the coincident DSM criteria group, there was a statistically higher prevalence of participants who perceived having mental health problems (35.1% vs 20.0%, *p* = 0.002), alcohol abuse (15.3% vs 7.14%, *p* = 0.028), and a history of psychiatric problems (44.2% vs. 28.0%, *p* = 0.001). In addition, this group reported a higher proportion of patients who had undergone previous treatment due to gambling problems (48.4% vs. 36.6%, *p* < 0.001), and gambling using slot machines (86.5% vs. 78.6%, *p* = 0.026).

**Table 2:** Clinical comparison of patients with coinciding and divergent DSM criterion.

	DSM5_A = Present DSM-IV_A = Present Coinciding; *n* = 1,822	DSM5_A = Present DSM-IV_A = Absent Divergent; *n* = 107	*p*	*| d |*
Previous treatments for GD; *%*	48.4	36.6	<0.001	0.24
Gambling: slot machines; *%*	86.5	78.6	0.026	0.21
Gambling: bingo; *%*	12.5	11.7	0.809	0.02
Gambling: lotteries; *%*	9.70	10.7	0.743	0.03
Gambling: casino; *%*	6.42	3.88	0.301	0.11
Gambling: cards; *%*	3.89	3.88	0.998	0.00
Gambling: sports or horse races; *%*	2.31	3.88	0.311	0.09
Gambling: internet; *%*	4.06	2.91	0.564	0.07
Gambling: other; *%*	1.98	3.92	0.181	0.11
Presence of secondary activity; *%*	21.7	22.2	0.896	0.01
Mental comorbidities at present; *%*	35.1	20.0	0.002	0.34
Smoker; *%*	74.8	66.0	0.051	0.19
Alcohol abuse; *%*	15.3	7.14	0.028	0.26
Substances abuse; *%*	10.3	9.18	0.712	0.04
Other addictive behaviors; *%*	8.12	6.06	0.546	0.08
Personal psychiatric history; *%*	44.2	28.0	0.001	0.34
Family psychiatric history; *%*	60.9	61.0	0.978	0.00
Age (years-old); mean (*SD*)	41.5 (12.8)	45.9 (15.1)	0.001	0.00
Age of onset (years-old); mean (*SD*)	35.5 (12.7)	41.5 (15.1)	<0.001	0.43
Duration of gambling probl. (years); mean (*SD*)	5.84 (6.25)	3.99 (4.30)	0.005	0.34
Maximum bets (euros); mean (*SD*)	940.6 (2,471.9)	747.4 (2,084.0)	0.460	0.08
Mean bets (euros); mean (*SD*)	185.4 (790.2)	154.5 (507.1)	0.753	0.05
Cumulate bets (euros); mean (*SD*)	9,860 (27,183)	14,444 (60,740)	0.178	0.10
Own income (euros); mean (*SD*)	1,198 (674)	1,390 (1,046)	0.009	0.22
Family income (euros); mean (*SD*)	2,100 (1,179)	2,361 (3,621)	0.120	0.10
Previous consultations for gambling; mean (*SD*)	0.80 (3.63)	0.49 (2.34)	0.431	0.10
Gambling severity-level: SOGS-total; mean (*SD*)	10.7 (2.89)	7.30 (2.56)	<0.001	**1.23^∗^**
Gambling severity-level: DSM_A-total; mean (*SD*)	7.54 (1.47)	4.00 (0.00)	<0.001	**3.41^∗^**
SCL-90: Somatization; mean (*SD*)	0.98 (0.81)	0.58 (0.60)	<0.001	**0.56^∗^**
SCL-90: Obsessive/compulsive; mean (*SD*)	1.17 (0.81)	0.76 (0.66)	<0.001	**0.55^∗^**
SCL-90: Interpersonal sensitivity; mean (*SD*)	1.07 (0.82)	0.60 (0.62)	<0.001	**0.65^∗^**
SCL-90: Depressive; mean (*SD*)	1.53 (0.90)	0.90 (0.71)	<0.001	**0.78^∗^**
SCL-90: Anxiety; mean (*SD*)	1.05 (0.82)	0.53 (0.55)	<0.001	**0.74^∗^**
SCL-90: Hostility; mean (*SD*)	0.94 (0.83)	0.49 (0.60)	<0.001	**0.62^∗^**
SCL-90: Phobic anxiety; mean (*SD*)	0.52 (0.68)	0.24 (0.53)	<0.001	0.46
SCL-90: Paranoid Ideation; mean (*SD*)	0.92 (0.75)	0.60 (0.70)	<0.001	0.44
SCL-90: Psychotic; mean (*SD*)	0.93 (0.75)	0.50 (0.56)	<0.001	**0.65^∗^**
SCL-90: GSI; mean (*SD*)	1.09 (0.69)	0.62 (0.53)	<0.001	**0.76^∗^**
SCL-90: PST; mean (*SD*)	48.0 (21.0)	32.8 (19.3)	<0.001	**0.75^∗^**
SCL-90: PSDI; mean (*SD*)	1.90 (0.59)	1.58 (0.50)	<0.001	**0.59^∗^**
TCI-R: Novelty seeking; mean (*SD*)	110.4 (13.9)	98.7 (11.9)	<0.001	**0.91^∗^**
TCI-R: Harm avoidance; mean (*SD*)	102.1 (17.1)	95.2 (16.3)	<0.001	0.42
TCI-R: Reward dependence; mean (*SD*)	99.6 (15.6)	102.2 (14.1)	0.120	0.17
TCI-R: Persistence; mean (*SD*)	109.8 (20.8)	114.1 (18.6)	0.050	0.22
TCI-R: Self-directedness; mean (*SD*)	125.2 (20.9)	140.5 (17.5)	<0.001	**0.80^∗^**
TCI-R: Cooperativeness; mean (*SD*)	131.4 (17.7)	138.1 (15.3)	<0.001	0.41
TCI-R: Self-Transcendence; mean (*SD*)	65.1 (15.3)	63.2 (14.6)	0.232	0.13

*SD: standard deviation. ^∗^Bold: moderate (|d| > 0.50) to high (|d| > 0.80) effect-size.*

The second part of the table contains the results comparing the two groups of the study (age, age of onset and duration of gambling problems, GD severity, psychopathological state, and personality traits). Overall, the group with coincident DSM criteria included younger patients, younger age of onset of gambling problems, and longer GD duration. In addition, this group presented higher GD severity (measured with the SOGS scale) and worse psychopathological state (higher mean scores on SCL-90-R scales).

## Discussion

This study analyzed whether the higher GD severity seen in treatment-seeking patients with GD who had committed gambling-related illegal acts is mediated through the DSM illegal acts criterion itself, or whether it can be better explained by a mediating role of other clinical features.

The main results of the present study indicated that 76.74% of the sample did not endorse the criminal acts criterion, while 23.26% presented it. Engagement in criminal behavior during the course of the disease has been associated with greater severity and worse prognosis, which yields implications for treatment interventions (Strong and Kahler, 2007; Turner et al., 2016; Mestre-Bach et al., 2018a,b). These findings are in line with the results of the present study, which indicate that patients with a history of criminal behavior have a higher level of GD severity compared to patients who do not endorse this criterion. The results also show that some personality characteristics were positively associated with the presence of criminal behavior while others were inversely associated. One example is the case of harm avoidance and self-transcendence, characteristics opposite to the profile of the patient who commits criminal acts. Comorbidity with other mental disorders may also affect the prognosis of the disease, often making it difficult to address the main disorder if secondary disorders have not been treated.

Some authors have indicated that the reduction in diagnostic criteria for GD in the new version of the manual would have an important impact on the prevalence ratio of the disease, and even more so in youth populations (Mitzner et al., 2011). However, this indication was not found in the present study, since the diagnosis of only 5.55% of the total sample shifts in the current version of the manual. Regarding the omission of the criterion of criminal acts, the authors promote this decision because of its low prevalence in the surveys carried out in the population. In our study, the criterion related to the commission of illegal acts supposes a compliance of 23.26% of the sample. Further studies have verified the limited usefulness of the item in diagnosis, considering it to serve more as an indicator of disorder severity than as an independent diagnostic criterion (Petry et al., 2013; Granero et al., 2014; Wejbera et al., 2017). Moreover, it has been suggested that GD-related criminal behaviors seldom take place in the absence of other GD criteria (Jiménez-Murcia et al., 2009).

However, the results in the present study suggest that the assessment of the illegal acts criterion might be clinically relevant, not so much to diagnose a patient with GD, but as an aid in measuring the severity and possible prognosis of the disorder, as well as in the decision of which therapeutic interventions to advocate. It is true that this criterion is important since many of the patients in which if the appearance of illegal acts in their diagnosis are involved in the legal system because they use all kinds of strategies to get the funding they need (theft, fraud, etc.). This is in line with the opinion of some authors who consider the illegal acts criterion to be essential for clinical practice (Mitzner et al., 2011). They suggest that the underreporting of criminal behaviors in a clinical assessment, possibly associated to the social desirability bias, may partially explain the low prevalence rates of this criterion, which was one of the motives for eliminating this criterion in the DSM-5 (Rash and Petry, 2016).

One of the most interesting findings in the present study was the high percentage of patients that endorsed the illegal acts criterion, where more than one third of women and more than half of the men indicated that they had participated in illegal acts to finance their gambling. Previous research has demonstrated that both sexes show common risk factors for criminal behavior, such as low education levels or substance abuse (Freudenberg et al., 2008; Herbst et al., 2016).

Finally, the data of this study indicate that the variables related to the financing of the gambling were not related to the commission of illegal acts. That is, financing or debts were not directly related to criminal conduct. These results contradict previous findings by asserting associations between debts and crime and suggesting that financial difficulties could increase the likelihood of GD related criminal behaviors, to find relief from financial hardship (Hoeve et al., 2014; Mestre-Bach et al., 2018b).

## Limitations and Future Research

The main limitation of the study is the lack of knowledge about the prevalence of compliance with each of the criteria necessary to perform the diagnosis of the disorder in both versions of the manual. Through this knowledge we could have studied more precisely what has been the change regarding the suppression of the criterion related to the commission of criminal acts in GD, in DSM-5. It would also have been very interesting to know the requirements by which the clinic is governed to establish the level of severity of the disorder and thus to be able to review in DSM-5 if the diagnostic modifications made have caused significant changes in the levels of severity of the disorder, taking into account the consequences they could cause at the clinical level. Future research should also assess GD-related crimes and their legal implications in depth. Moreover, being that data were obtained via a face-to-face clinical interview, they could be subject to social desirability bias, and, consequently, false negatives are likely due to unreported criminal behaviors. Finally, our sample was only made up of a clinical population, specifically patients who sought treatment for GD. Future studies would benefit from also including gamblers from the general population, to obtain a more exhaustive perspective of different existing phenotypes.

### Clinical Implications

In terms of clinical implications, having a psychological and behavioral profile of patients diagnosed with GD, taking into account the compliance of the DSM criterion of commission of criminal acts could be very beneficial to inform the treatment and the mode of intervention. This description of both profiles would be very interesting considering the salient aspects that would emerge in the diagnosis, using both versions of the manual and checking if taking into account or not said criterion could influence the patient’s clinic.

The main treatment used in patients diagnosed with GD relies on cognitive behavioral therapy (CBT) based on live exposure techniques. Response prevention and the control of stimuli are intended to provoke the desire to gamble and allow the patient to learn to resist and control those desires progressively (Oei et al., 2010, 2017; Rash and Petry, 2014; Jiménez-Murcia et al., 2015). Patients who engage in criminal behavior may require a more extensive type of intervention. It is also very important to consider the risk of suicide, since the prevalence of suicidal attempts among GD patients endorsing the illegal acts criterion has been found to be higher (Potenza et al., 2000; Ledgerwood et al., 2005).

## Conclusion

In conclusion, the results obtained in the present study provide an increased insight to GD patients, their different personality characteristics and psychopathology in relation to the DSM illegal acts criterion. Future studies focusing on admission processes and the design of individual treatments for GD patients is recommended, in which the results presented in this study could serve a beneficial role.

## Ethics Statement

The present study was carried out in accordance with the latest version of the Declaration of Helsinki. The Bellvitge University Hospital Ethics Clinical Research Committee approved the study. Signed, informed consent was obtained from all patients.

## Author Contributions

SJ-M, FF-A, AS, AH, RS, and JMM contributed to the development of the study concept and design. RG performed the statistical analysis. SJ-M, RG, FF-A, AF, AH, GM-B, TS, LM, NA, MG-P, AP-G, ZA, MB, M-TT-N, ÀC, and EC conducted the experiment and wrote the manuscript.

## Conflict of Interest Statement

The authors declare that the research was conducted in the absence of any commercial or financial relationships that could be construed as a potential conflict of interest.
